# Humanistic and financial burden experienced by informal care partners of patients with mild cognitive impairment due to Alzheimer's disease and dementia due to Alzheimer's disease: Results from real‐world survey

**DOI:** 10.1002/alz70858_100779

**Published:** 2025-12-25

**Authors:** Riya Arora, Niels Juul Brogaard, Sharon Cohen, Soeren Mattke, Sarah Cotton, Chloe Walker

**Affiliations:** ^1^ Novo Nordisk Service Centre India Pvt Ltd, Bangalore, India; ^2^ Novo Nordisk A/S, Søborg, Denmark; ^3^ Toronto Memory Program, Toronto, ON, Canada; ^4^ University of Southern California, Los Angeles, CA, USA; ^5^ Adelphi Real World, Bollington, United Kingdom

## Abstract

**Background:**

The burden of care for patients with Alzheimer's disease (AD) often falls to family members. We aimed to explore burden experienced by informal care partners of patients with mild cognitive impairment (MCI) and dementia (both due to AD), across disease severity stages.

**Method:**

Data were drawn from the Adelphi Real World AD Disease Specific Programme™, a cross‐sectional survey of physicians, their patients with MCI due to AD or dementia due to AD (clinically diagnosed or biomarker confirmed) and care partners in Canada, France, Germany, Italy, Spain, the United Kingdom, Japan and the United States, between December 2022 – March 2024. Physicians were asked to report data on Mini‐Mental State Examination (MMSE) scores for their next nine consulting patients, followed by one patient with a biomarker‐confirmed MCI due to AD diagnosis. Care partners self‐reported their care activities, and impact of caring responsibilities on their lifestyle and health. Disease severity was categorised using the patient's current MMSE score. Analyses were descriptive.

**Result:**

Overall, 829 physicians provided data for 4202 MCI/dementia due to AD patients (10.3% with an MMSE of 26‐30, 37.8% 21‐25, 46.7% 11‐20, 5.3% 0‐10); 484 care partners self‐reported data. Care partners had a mean (standard deviation) age of 61.6 (14.7) years and 64.8% were female. Most were the patients’ partner/spouse (45.9%) or child (37.0%).

Care partners reported helping patients take medication when required (30.0% of care partners for patients with an MMSE of 26‐30; 48.8% 21‐25; 71.7% 11‐20; 87.1% 0‐10), remembering/making appointments (56.7%; 47.5%; 66.1%; 77.4%) and preparing food (30.0%; 48.8%; 65.0%; 87.1%). Care partners reported having less time to themselves (27.6%; 38.2%; 50.6%; 62.1%), decreased social activities (17.2%; 22.4%; 28.6%; 27.6%) and neglecting their own health (6.9%; 5.9%; 14.7%; 24.1%). Care partners also reported experiencing stress (28.1%; 43.1%; 40.9%; 71.0%), anxiety (34.4%; 32.5%; 35.4%; 41.9%) and back pain (34.4%; 33.1%; 31.5%; 32.3%).

**Conclusion:**

Care partners of patients with MCI/ dementia due to AD experience burden across various aspects of their life, often neglecting personal time and their health as a result of caregiving. Ensuring early interventions to slow disease progression is key to reducing care partner burden.

